# The variability of PCDD/F concentrations in the effluent of wastewater treatment plants with regard to their hydrological environment

**DOI:** 10.1007/s10661-017-5794-9

**Published:** 2017-01-31

**Authors:** Magdalena Urbaniak, Edyta Kiedrzyńska, Adam Grochowalski

**Affiliations:** 1 European Regional Centre for Ecohydrology of the Polish Academy of Sciences, Tylna 3, 90-364 Lodz, Poland; 2 Department of Applied Ecology, Faculty of Biology and Environmental Protection, University of Lodz, Banacha 12/16, 90-237 Lodz, Poland; 3 Department of Chemical Engineering and Technology, Department of Chemical Engineering and Technology, Krakow University of Technology, Warszawska 24, 31-155 Krakow, Poland

**Keywords:** PCDDs/Fs, Wastewater, WTP, Effluents

## Abstract

The occurrence, concentrations, patterns, and loads of 17 2,3,7,8-substituted congeners of polychlorinated dibenzo-*p*-dioxins (PCDDs) and polychlorinated dibenzofurans (PCDFs) were determined in treated wastewater collected at the outlets from 14 wastewater treatment plants (WTPs), divided into three size categories. The analysis also considered the effect of wastewater outflow on the final concentrations of PCDDs/Fs, as the samples were collected during flooding and stable hydrological conditions. None of the studied WTPs were found to completely remove toxic congeners of PCDDs/Fs from wastewater: the PCDD concentrations in the outgoing effluent during stable wastewater flow ranged from 2.99 to 177.19 pg/L, PCDFs from 6.05 to 51.30 pg/L, and the Toxic Equivalent (TEQ) was between 0.94 and 4.87 pg/L. The results from high wastewater flow were less diversified, ranging from 5.04 to 8.85 pg/L for PCDDs, from 11.47 to 32.33 pg/L for PCDFs and from 2.00 to 4.65 pg/L for TEQ. The smallest WTPs demonstrated the highest average total and TEQ concentrations due to limited volume, short retention time and outdated technology, and, hence, insufficient treatment efficiency. The largest WTPs have the potential to substantially affect the quality of river water, as despite being associated with the lowest concentrations, they released much greater volumes of treated wastewater and, hence, the greatest amounts of analyzed compounds. Elevated TEQ values were observed at high flow in all WTP size categories indicating the impact of high and stable wastewater flow on the concentrations of the observed PCDDs/Fs.

**Electronic supplementary material:**

The online version of this article (doi:10.1007/s10661-017-5794-9) contains supplementary material, which is available to authorized users.

## Introduction

The global population has grown rapidly from approximately 5.3 billion in 1992 (UNEP 2012) to about 6.97 billion in 2011 (UN Population Division 2011), and according to the United Nation projections, the numbers will reach over eight billion in 2030 and nine billion in 2050. The effect of such rapid population growth is reflected in increasing consumption of global water and consequential production of wastewater. The US EPA reports (2000; 2004) and Carey and Migliccio (2009) note that influent wastewater flow in the USA is predicted to rise from 100,000,000 m^3^/day in 1996 to 170,000,000 m^3^/day in 2025. This is also the case of Poland, where the total amount of treated wastewater increased by about 38% between 1980 and 2007 (Wałęga et al. 2009).

The accession of Poland to the European Union and the consequent implementation of European water management directives, with their implications for inland water quality (Mostert 2003) and European water and wastewater policy (Kiedrzyńska et al. 2014a), have obliged the country to improve wastewater management by 2015. The Water Framework Directive (WFD 2000/60/EC), for example, requires good inland water status to be achieved and for certain priority substances to be managed, through integrated river basin management. The Nitrates Directive (ND 91/676/EEC), in turn, promotes various nitrogen-reducing management practices in the agricultural sector, and the Urban Wastewater Treatment Directive (UWWTD 91/271/EEC) regulates the collection and treatment of wastewater in urban areas (Kiedrzyńska et al. 2014a).

However, in the case of PCDDs/Fs, which are the most toxic organochlorine compounds occurring in the water environment, the regulations concerning their concentrations and limits in the wastewater effluents discharged into the river recipients remain insufficient. For example, one of the most important Polish regulations in the field of water policy, the Water Law (OJ 2001 No. 115, item 1229, act of July 19 2001, Water Law) does not regulate the limits for the concentration of toxic PCDDs/Fs in either wastewater effluents or in surface water. Such limits can be found in EC Regulation No. 166/2006 of the European Parliament and of the Council of 18 January 2006 concerning the establishment of a European Pollutant Release and Transfer Register and amendments to Council Directives 91/689/EEC and 96/61/EC, which establish a threshold of 0.0001 kg per year for releases of PCDD + PCDF (as TEQ) from municipal WTPs into the water column, for a population equivalent equal to 100,000. However, smaller WTPs are not inspected with regard to PCDD/F release.

Intensive studies on the occurrence of PCDDs/Fs in untreated wastewater and sewage sludge during recent decades have revealed very high concentrations, with a predominance of highly chlorinated congeners (Hagenmayer et al. 1986; Rappe et al. 1989; Broman et al. 1990; Näf et al. 1990; Sewart et al. 1995; Alcock and Jones 1997; McLachlan et al. 1996; Rappe et al. 1998; Eljarrat et al. 1999, 2003; Koch et al. 2001; Dudzińska and Czerwiński 2002; Oleszek-Kudlak et al. 2005). Nevertheless, little is known of their fate during wastewater treatment and their final concentrations in the treated effluents (Rogers 1996). Some previous studies have demonstrated that the wastewater purification process results in increased concentrations of lower chlorinated, more toxic PCDD/F congeners in outgoing wastewater (Sztamberek-Gola et al. 2003; Oleszek-Kudlak et al. 2005) and so emphasize the need for wastewater effluent monitoring.

Hence, the aim of this study was to determine the occurrence, concentrations, patterns, and loads of 2,3,7,8-substituted congeners of PCDD and PCDF in treated wastewater discharged from 14 WTPs, divided into three size categories, all of which are located in one river catchment. The effect of wastewater outflow on the final concentrations of PCDDs/Fs was also examined, as the treated wastewater samples were collected during spring flood and during stable hydrological conditions in late summer.

## Materials and methods

### Study area and WTP descriptions

**Fig. 1:**
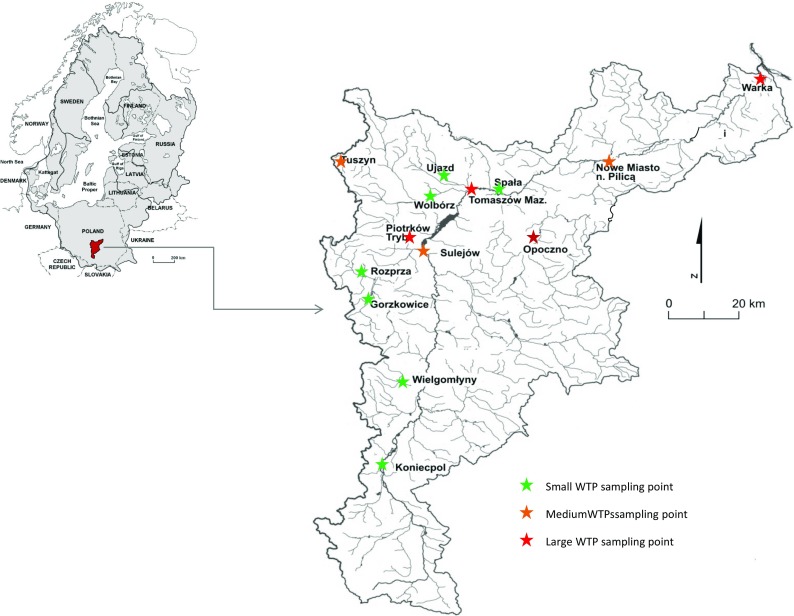
Location of the WTP sampling points located in the Pilica River catchment (central Poland)

The studied WTPs are located in the Pilica River catchment (central Poland), which has a total catchment area of 9258 km^2^. The complete Pilica river length is 342 km and is the longest left-hand tributary of the Vistula River, as well as one of its most significant tributaries, entering the Vistula at 457 km along the river course (Urbaniak et al. 2012a, b; 2014; Kiedrzyńska et al. 2014a) (Fig. 1). The river catchment is predominantly agricultural (60% of its total area) and forest (31% of its total area).

The wastewater system usage in the Pilica River catchment supports from 51.3 to 70.5% of the human population, depending on the region, with a mean value for the whole catchment of 59%. However, of all the 143 WTPs located in its catchment, only 43.6% are equipped with advanced nutrient removal technology (Kiedrzyńska et al. 2014b).

The 14 WTPs selected for the present study were divided into three size categories according to the number of adults whose wastewater they can process (population equivalent—p.e.): small (0–1999 p.e.), medium (2000–9999 p.e), and large (15,000–99,999 p.e.). The study included seven small WTPs, three medium-sized WTPs, and four large WTPs (Table 1). Five WTPs, those in Koniecpol, Sulejów, Tomaszów Mazowiecki, Nowe Miasto, and Warka, released the treated wastewater directly into the Pilica River, while the remainder was discharged into the Pilica River tributaries. All the studied WTPs utilize secondary (mechanical-biological) treatment.

**Table 1:** The characteristics of the studied WTPs located in the Pilica River catchment (central Poland)

WTP category	WTP location	Population equivalent	Treated wastewater outflow—average [m^3^/day]	Treated wastewater outflow—high flow [m^3^/day]	Treated wastewater outflow—stable flow [m^3^/day]
Small WTPs (0–1999 p.e.)	Koniecpol	600	100	n.a.	n.a.
	Rozprza	500	107	331	89
	Spała	350	130	132	115
	Wielgomłyny	1000	200	89	42
	Gorzkowice	700	224	577	201
	Wolbórz	800	241	490	290
	Ujazd	1500	300	420	406
Medium WTPs (2000–9999 p.e.)	Tuszyn	4000	640	1086^a^	766
	Sulejów	7500	870	1264^b^	1572
	Nowe Miasto	2583	1000	410	536
Large WTPs (15000–99,999 p.e.)	Opoczno	75,000	5127	9307	3932
	Warka	99,000	9900	4410	1757
	Tomaszów Mazowiecki	80,000	10,050	11,432	7624
	Piotrków Trybunalski	80,000	14,541	25,470	19,020

*n.a.* data not available, *p.e.* population equivalent

^a^Data from 27 May 2010

^b^Data from 25 May 2010

### Sampling

Two 5 L samples of treated wastewater were collected from each WTP. The samples were taken at high wastewater flow (19-20.05.2010) and at stable wastewater flow (26-27.09.2010) (Fig. 1; Table 1). The samples were collected directly from the wastewater outflow into the Pilica River or its tributaries into amber containers and transported to the laboratory in a car fridge at a temperature of 4 °C.

### Analysis of PCDD/F concentration

The PCDD/F analyses were performed in an accredited laboratory at the Krakow University of Technology, Krakow, Poland, according to Urbaniak et al. (2014). Briefly, 2.00 L samples of treated wastewater were spiked with 60.0 pg of 17 ^13^C-labeled PCDDs/Fs (NK-LCS-G and WP-LCS, Wellington Laboratories) dissolved in 1 mL methanol. Toluene was then used for liquid/liquid extraction. The obtained extract was placed in the bottom of a tube sealed by a polyethylene semipermeable membrane and cleaned overnight with 100 mL hexane. The obtained dialysate was then cleaned using a silica gel column filled with 44.0% sulphuric acid and alumina (U.S. EPA Method 1613 1994). After this step, 20.0 μL of precision and recovery solution prepared in nonane was added to the obtained extracts (EPA1613 ISS mix of 200 ng/mL of ^13^C_12_-1,2,3,4-TCDD and ^13^C_12_-1,2,3,7,8,9-HxCDD).

A Thermo Scientific GCQ-1100/Trace2000 Isotope dilution gas chromatography-tandem mass spectrometry (ID-GC/MS-MS) system and Xcalibur data acquisition and analysis software were used to determine the quantities of seven PCDDs and ten PCDFs in the water. For proper separation of congeners, a 30.0 m × 0.250 mm i.d. DB5MS J&W capillary column with 25-μm film and DB17 30.0 m × 0.250 mm i.d. DB5MS J&W capillary column with 25-μm film was used. A 2.50 μL sample volume was injected into the SSL injector at a temperature of 260 °C.

The GC oven was programmed with the following sequence: the initial temperature of 130 °C was maintained for 3 min before being ramp by 50 °C/min to 180 °C, then again by 2 °C/min to 270 °C, and then by 20 °C/min to 300 °C, where it was held for 5 min. The resulting uncertainty was expressed as extended measurement uncertainty for *k* = 2 at a confidence level of 95% (Urbaniak et al. 2014).

### Quality assurance/quality control

The Laboratory for Trace Organic Analyses at the Krakow University of Technology, Poland, is a member of the Interlaboratories for Dioxins Circuit organized by the Interuniversity Consortium “Chemistry for the Environment” and LabService Analytica S.r.l. The internal reference materials were used to properly validate the analytical method; the analytical laboratory is fully accredited with accreditation no. AB 749.

Quantification was achieved by the internal standard method using certified calibration standards. A method blank, a matrix spike, and replicate samples were used in each analytical batch. Moreover, in order to assess the artifacts, a reagent blank was used, while duplicate analyses were used to verify the precision. The sample spikes were used to further confirm accuracy. Recoveries were estimated using samples spiked with PCDDs/Fs and were found to range from 64 to 122%, while LOD ranged from 0.22 pg/L for TeCDD/TeCDF to 2.80 pg/L for OCDD/OCDF.

All glassware and bottles used in the field and laboratory were cleaned with detergent then rinsed with ultrapure water and heated at 450 °C overnight. Before use, the glassware was rinsed with acetone and then hexane. Similarly, the Teflon containers used in the field were cleaned with detergent, rinsed with ultrapure water, and rinsed with acetone and hexane before application.

### Analysis of PCDD/F load

To calculate the daily loads of PCDDs, PCDFs, and TEQ, the daily wastewater outflows (m^3^/day) measured at each studied WTPs during conditions of flooding and stable hydrology (Table 1) were multiplied by the total and TEQ concentrations (pg/L) of the PCDDs and PCDFs. The obtained loads were depicted as microgram of total PCDDs, PCDFs, or TEQ per day.

## Results and discussion

The National Program of Urban Wastewater Treatment (Poland) (KZGW 2013) requires any agglomeration producing wastewater with a pollution load equivalent to the amount of wastewater generated by 2000 adults (p.e. more than 2000) to be equipped with a wastewater collection and treatment system appropriate to local conditions and needs. Between 2003 and 2015, 1700 WTPs were designed for operation in 1577 agglomerations of more than 2000 p.e., while a further 29 WTPs are intended for agglomerations of less than 2000 p.e. (Smołka 2008; KZGW 2013).

Such a rapid growth of WTPs should lead to more effective removal of contaminants from inflowing wastewater, thereby producing an effluent which is environmentally safe. Nevertheless, the available literature data indicates that conventional wastewater treatment systems are not able to sufficiently remove hydrophobic contaminants, and these have adverse effects on the receiving water ecosystem (Pham et al. 1999; Blanchard et al. 2001; Katsoyiannias and Samara 2004; Bergqvist et al. 2006; Joss et al. 2006; Katsoyiannis and Samara 2007; Cirja et al. 2008; Carey and Migliccio 2009; Jelic et al. 2011; Grover et al. 2011; Saffari and Saffari 2013; Urbaniak et al. 2014; Kiedrzyńska et al. 2014b). This insufficiency results in the presence of organic compounds in river water worldwide (Gotz et al. 1994; Camusso et al. 2000; Kakimoto et al. 2006; Chen et al. 2008; Chi et al. 2011; Minomo et al. 2011), including Polish water bodies (Kowalewska et al. 2003; Wolska et al. 2003; Urbaniak et al. 2012a, b; 2014).

For many years, quantification of wastewater effluents and receiving river water pollution was restricted to monitoring biochemical oxygen demand, chemical oxygen demand, nitrogen and phosphorus concentrations, and total suspended solids (Cirja et al. 2008). However, as shown in our earlier study, significant concentrations of nitrogen and phosphorus which exceed the allowable limits for the type of WTP may be present in treated wastewater, with the highest values in the smallest WTPs (Kiedrzyńska et al. 2014b). A similar situation was observed by Urbaniak et al. (2014) in the case of PCDDs/Fs. All WTPs studied by Urbaniak et al. (2014) were found to discharge toxic PCDD/F and dl-PCB compounds into their receiving rivers. This can be attributed to insufficient regulation of the discharge of toxic congeners of PCDDs/Fs by municipal WTPs: the existing regulations only apply to municipal WTPs with a p.e. of 100,000 which exclude the studied WTPs located in the Pilica River catchment. This, together with the increasing number of municipal WTPs (KZGW 2013) and increases in the concentrations of the lower chlorinated, and hence more toxic, PCDDs/Fs in WTP outlet water, as noted by Sztamberek-Gola et al. (2003) and Oleszek-Kudlak et al. (2005), may result in poorer quality of the receiving waters. Consequently, the results obtained in the present study provide hitherto missing information about the concentrations, patterns, and loads of PCDDs/Fs in treated wastewater discharged into the Pilica River and its tributaries under various hydrological conditions, which are reflected in the composition of the treated wastewater outflow.

### The occurrence and changes in total and TEQ concentrations at high and stable wastewater flow according to WTP size categories

**Fig. 2:**
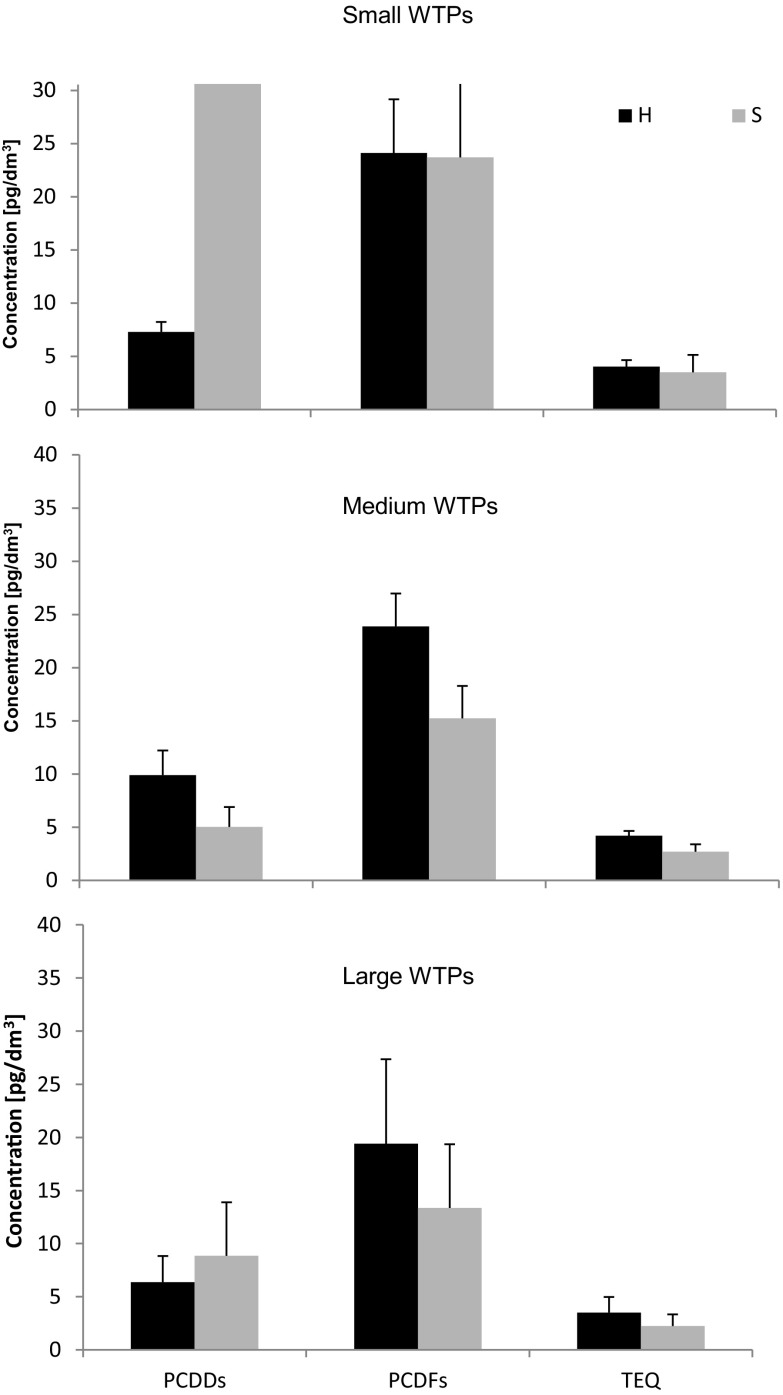
Changes in the average PCDD, PCDF, and TEQ concentrations at high (H) and stable wastewater flow (S) (the Pilica River catchment, central Poland)

The total and TEQ concentrations of seven 2,3,7,8-substituted PCDDs and ten 2,3,7,8-substituted PCDFs are given in Table 2 and Fig. 2.

**Table 2:** Concentrations of the sum of 7 2,3,7,8-substituted PCDDs, sum of 10 2,3,7,8-substituted PCDFs, and TEQ in WTPs effluents collected at high (H) and stable wastewater flow (S) (the Pilica River catchment, central Poland)

WTP category	WTP location	H			S		
		PCDD [pg/L]	PCDF [pg/L]	TEQ [pg TEQ/L]	PCDD [pg/L]	PCDF [pg/L]	TEQ [pg TEQ/L]
Small WTPs (0–1999 p.e.)	Koniecpol	6.32	18.11	3.17	9.99	23.99	5.05
	Rozprza	7.46	23.66	4.26	8.73	10.76	1.66
	Spała	8.54	27.60	4.73	7.50	17.50	2.69
	Wielgomłyny	7.72	32.33	4.30	9.20	29.50	4.76
	Gorzkowice	5.94	19.08	3.40	50.30	33.00	4.32
	Wolbórz	8.19	26.45	4.65	173.19	51.30	4.87
	Ujazd	6.86	21.60	3.77	2.79	6.92	1.13
Medium WTPs (2000–9999 p.e.)	Tuszyn	10.61	23.13	4.36	2.99	12.42	1.98
	Sulejów	10.22	27.29	4.56	6.64	18.48	3.27
	Nowe Miasto	6.43	21.20	3.69	5.50	14.80	2.90
Large WTPs (15000–99,999 p.e.)	Opoczno	6.86	21.36	3.54	10.90	20.70	3.57
	Warka	3.39	11.47	2.00	5.20	13.60	2.16
	Tomaszów Mazowiecki	9.35	29.66	5.48	4.30	13.10	2.40
	Piotrków Trybunalski	5.88	15.12	2.97	15.01	6.05	0.93

*p.e.* population equivalent

The highest concentration diversity was noted during stable wastewater flow for the sum of seven PCDD congeners, ranging from 2.99 to 173.19 pg/L. Very high average values were also observed for small WTPs (37.40 pg/L) as an effect of increased PCDD concentration in the Wolbórz WTP, while these values were several times lower for medium and large WTPs (5.04 and 8.85 pg/L, respectively) (Table 2). In contrast, the samples taken during high flow showed less diversity in PCDD concentrations, ranging from 3.39 to 10.61 pg/L, with the lowest average concentration noted for large WTPs (6.37 pg/L) and the highest for medium WTPs (9.09 pg/L) (Table 2; Fig. 2).

Similarly, PCDF values were more diverse in samples collected at stable flow, ranging from 6.05 to 51.30 pg/L, than high wastewater flow, ranging from 11.47 to 32.33 pg/L. In both sampling periods, mean PCDF values were observed to decrease as the WTP size increased: from 24.12 pg/L in small WTPs, through 23.87 pg/L in medium WTPs to 19.40 pg/L in large WTPs during high flow, and from 23.72 pg/L in small, through 15.23 pg/L in medium-sized to 13.36 pg/L in large WTPs during stable flow. In addition, a decline was also observed between the first and second samples for particular WTP categories: a decrease from 24.12 to 23.72 pg/L in small, from 23.87 to 15.23 pg/L in medium, and from 19.40 to 13.16 pg/L in large WTPs (Table 2; Fig. 2). In addition, the mean total PCDF values were found to be higher than those of the PCDDs among the small, medium, and large WTPs (Fig. 2). This could be attributed to the lower water solubility of the PCDD congeners than the PCDFs, reflected as the octanol-water partition coefficient (*K*_OW_) (Shiu et al. 1988). Thuan et al. (2011) report that the percentage of dissolved PCDFs is higher than that of dissolved PCDD homologues in water samples characterized by small amount of suspended particulates, such as groundwater.

The TEQ concentrations ranged between 2.00 and 4.65 pg TEQ/L in samples collected during high flow and from 0.93 to 4.87 pg TEQ/L in samples taken at stable flow: both of the highest values being noted in the same small WTP in Wolbórz. In terms of average TEQ, the highest concentrations were noted for small (3.50 pg TEQ/L) and medium WTPs (4.20 pg TEQ/L) during stable and high flow, respectively. During stable flow, average TEQ concentration was found to decline as WTP size increased (3.50, 2.72, and 2.26 pg TEQ/L for the small, medium, and large WTPs, respectively). A decrease was also observed between first and second samplings for particular WTP sizes—a decrease from 4.04 to 3.50 pg TEQ/L in small WTPs, from 4.20 to 2.72 pg TEQ/L in medium WTPs, and from 3.50 to 2.26 pg TEQ/L in large WTPs (Table 2; Fig. 2).

The obtained total and TEQ concentrations are several times higher than those found in the very scarce studies which have been performed on treated wastewater. Data presented by Sztamberek-Gola et al. (2003) and Oleszek-Kudlak et al. (2005), obtained on the basis of three WTP analyses, revealed total and TEQ concentrations within the range from 107.26 to 219.19 pg/m^3^ for PCDDs, from 201.75 to 736.50 pg/m^3^ for PCDFs, and from 14.70 to 116.40 pg I-TEQ/m^3^ for TEQ. Moreover, the authors observed increased PCDD and PCDF concentrations to be related to increased daily wastewater flow: the lowest values were noted in effluents from the smallest WTP, with a daily flow of 20,000 m^3^, whereas samples coming WTPs with twice the flow (40,000 and 45,000 m^3^) were found to have concentrations about two times higher. This is probably due to the greater input of toxic industrial wastewater in the case of the larger WTP. However, the opposite tendency was observed in our findings, with the lower total and TEQ concentrations in the largest WTPs with the highest flow. This is probably related to the insufficient treatment of wastewater in small WTPs, partly because of not only the limited volume and short retention time of wastewater in the WTP but also the use of outdated technology in some cases, as demonstrated in Kiedrzyńska et al. (2014b) with regard to nitrogen and phosphorus removal.

### Changes in PCDD/F patterns with regard to WTP size categories at high and stable wastewater flows

The occurrence of PCDDs/Fs in inflowing wastewater causes considerable problems for the WTPs, because conventional biological and chemical processes are insufficient for their removal. What is more, scarce data exists to explain how wastewater treatment affects the behavior and fate of PCDDs/Fs. Since PCDDs/Fs have a very high sorption potential (Mackay et al. 2006), they are expected to partition into the sewage sludge part of the wastewater during treatment processes. In addition, as the majority of treatment processes employ volatilization at some stage, the low volatilization potentials of PCDDs/Fs reduce their loss (Oleszek-Kudlak et al. 2005).

**Fig. 3:**
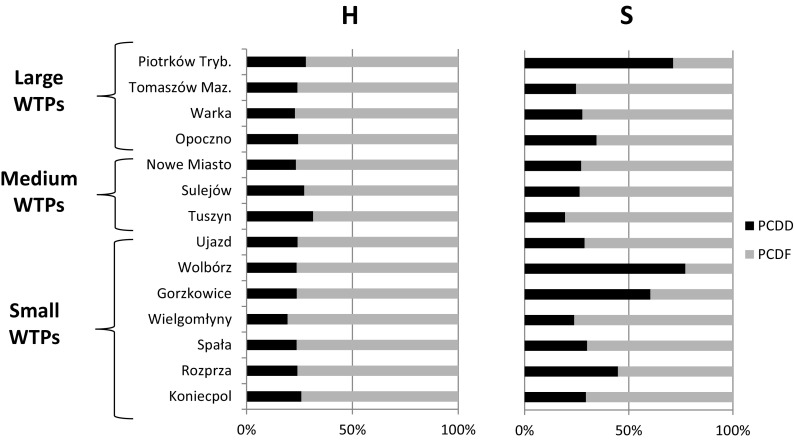
Contribution [%] of PCDDs and PCDFs in the total PCDD/F concentrations at high (H) and stable wastewater flow (S) (the Pilica River catchment, central Poland)

Oleszek-Kudlak et al. (2005) and Sztamberek-Gola et al. (2003) note that wastewater treatment affects the fate of PCDDs/Fs, with increased amounts of congeners with lower degrees of chlorination, and hence, greater toxicity, in the outlet effluents. As a consequence, the I-TEQ (International TEQ) concentrations are more than five times higher in the outgoing treated effluent than the incoming wastewater. Moreover, the authors report a predominance of PCDFs over PCDDs in the outgoing effluents, as noted in the present study, where at high wastewater flow, PCDD levels were only a fifth to a third of the PCDF levels (Fig. 3), with the average PCDDs content being 23.46, 27.32, and 24.77% in the small, medium, and large WTPs, respectively. A lower PCDD content was also observed in aqueous samples by Thuan et al. (2011).

**Fig. 4:**
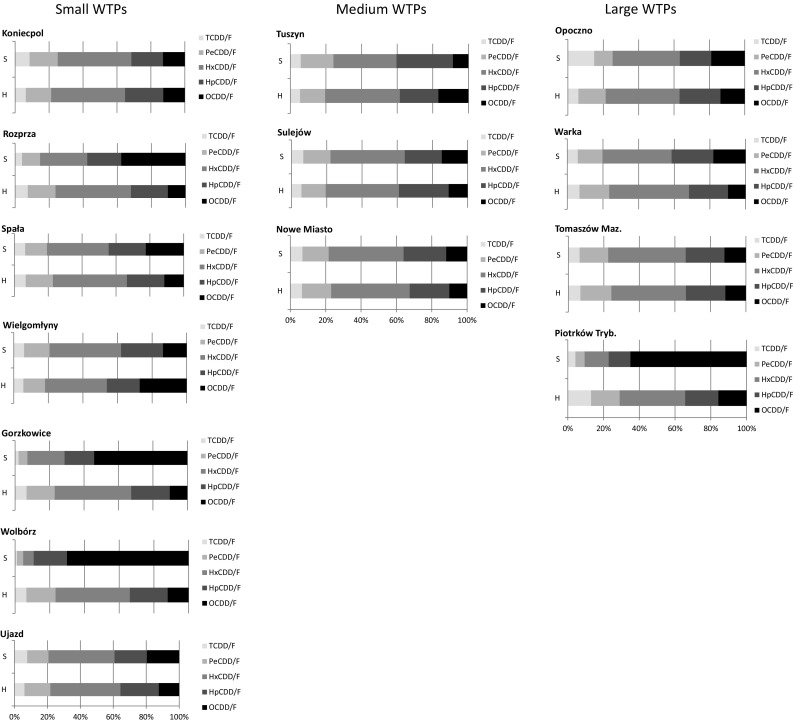
Contribution of tetra- (TCDD/F), penta- (PeCDD/F), hexa- (HxCDD/F), hepta- (HpCDD/F), and octa- (OCDD/F) congeners in the total PCDD/F content at high (H) and stable wastewater flow (S) (the Pilica River catchment, central Poland)

For the samples collected at stable wastewater flow, three WTPs, Gorzkowice and Wolbórz (small WTPs) and Piotrków Trybunalski (large WTP), showed an opposite tendency with higher levels of PCDDs being present (60.38, 77.15, and 71.27%, respectively). However, the remaining WTPs showed a similar trend during the flooding season, with the lower PCDD content ranging from 19.40 to 44.79% (Fig. 2). The high contribution of PCDDs in the Gorzkowice, Wolbórz, and Piotrków Trybunalski WTPs was mainly generated by enhanced concentrations of OCDD/F congeners amounting to 54.14, 70.07, and 64.96%, respectively (Fig. 4; Table 1S). These elevated OCDD/F congener contents observed in these three WTPs can be attributed to the insufficient treatment of the inflowing wastewater due to WTP maintenance performed during the second sampling: the treatment capacity of the given WTPs radically decreased as a consequence, affecting the quality of the outgoing effluents, which have similar profiles to the input to the WTP (Oleszek-Kudlak et al. 2005). At the second sampling, these WTPs released very high concentrations of total suspended particulates, as high as 338.84, 847.65, and 135.66 mg/L in Gorzkowice, Wolbórz, and Piotrków Trybunalski, respectively; while the concentrations noted at the first sampling were several times lower, amounting to 6.40, 32.20, and 6.60 mg/L. Such high divergence in the total suspended particulates highlights the difficulty of maintaining the proper purification efficiency in the given WTPs.

Similar results were obtained by Rappe et al. (1998) who note that effluent containing the most particulates had the highest values for the higher chlorinated PCDDs/Fs, with an OCDD concentration 90 times higher than the median value for all the samples.

Among other congeners, elevated contributions of up to 45% were noted for HxCDD/F (Fig. 3). Low percentages of highly toxic TCDD/F and PeCDD/F congeners were observed, ranging from 1.11 to 14.87 and from 3.52 to 18.36%, respectively (Fig. 3). The content of HpCDD/F congeners was slightly higher and ranged from 12.20 to 31.73% (Fig. 4; Table 1S). All of these homologue groups demonstrated similar patterns to those reported by Oleszek-Kudlak et al. (2005) and Sztamberek-Gola et al. (2003).

The obtained results showed also the increased contribution of PCDDs in samples collected at stable flow in comparison to those taken at high wastewater flow. The only exception to this rule was Tuszyn WTP (a medium WTP), where a 12% decrease was detected (Fig. 3). This increase during stable flow can be related to the lower contribution of diffuse pollution coming from the Pilica River catchment. This relationship was previously confirmed by Urbaniak et al. (2014), who note strong correlations as high as 0.98 between river water and treated wastewater sampled at flooding, while weaker, often insignificant, correlations were observed during stable water flow. These results indicate that during flooding, the river water and treated wastewater were almost identical in term of congener pattern and the most probable reason of such situation is the impact of diffuse sources of pollution occurring as a result of pollutant runoff from the catchment surface. The increase in wastewater volume during the flooding season (see Table 1) may further affect the purification process, mainly in terms of wastewater storage, during particular treatment steps (Clara et al. 2005).

### Changes in PCDD/F loads among WTP size categories at high and stable wastewater flow

In addition to its impact on the concentrations and patterns of PCDDs/Fs, the volume of wastewater also contributes to the levels of micropollutants released via effluents to the river recipients. The obtained results demonstrate the load of PCDDs, PCDFs, and TEQ increase with WTP size, with the lowest values being identified for the smallest WTPs in Wielgomłyny and Rozprza and the highest for the largest WPTs in Piotrków Trybunalski and Tomaszów Mazowiecki (Table 3).

**Table 3:** Loads of PCDDs, PCDFs, and TEQ through effluents at high (H) and stable wastewater flow (S) (the Pilica River catchment, central Poland)

WTP category	WTP location	H			S		
		PCDD [μg/day]	PCDF [μg/day]	TEQ [μg/day]	PCDD [μg/day]	PCDF [μg/day]	TEQ [μg/day]
Small WTPs (0–1999 p.e.)	Koniecpol	–	–	–	–	–	–
	Rozprza	2.47	7.83	1.41	0.78	0.96	0.15
	Spała	1.13	3.64	0.62	0.86	2.01	0.31
	Wielgomłyny	0.69	2.88	0.38	0.39	1.24	0.20
	Gorzkowice	3.43	11.01	1.96	10.11	6.63	0.87
	Wolbórz	4.01	12.96	2.28	50.23	14.88	1.41
	Ujazd	2.88	9.07	1.58	1.13	2.81	0.46
Medium WTPs (2000–9999 p.e.)	Tuszyn	11.52	25.12	4.73	2.29	9.51	1.52
	Sulejów	12.92	34.49	5.76	10.44	29.05	5.14
	Nowe Miasto	2.64	8.69	1.51	2.95	7.93	1.55
Large WTPs (15000–99,999 p.e.)	Opoczno	63.85	198.80	32.95	42.86	81.39	14.04
	Warka	14.95	50.58	8.82	9.14	23.90	3.80
	Tomaszów Mazowiecki	106.89	339.07	62.65	32.78	99.87	18.30
	Piotrków Trybunalski	149.76	385.11	75.65	285.49	115.07	17.69

*p.e.* population equivalent

The total load released from all small WTPs at high flow amounted to 14.61, 47.39, and 8.24 μg/day for PCDDs, PCDFs, and TEQ, respectively; at stable flow, PCDD loads increased to 63.49 μg/day (an increase of 335%), while PCDFs and TEQ declined to 28.53 (a decrease of 40%) and to 3.40 μg/day (a decrease of 59%), respectively (Table 3).

The total loads obtained for medium WTPs also decreased between the samplings. The values fell from 27.08 to 15.68 μg/day (a decrease of 42%) for PCDDs, from 68.31 to 46.50 μg/day (a decrease of 32%) for PCDFs, and from 12.01 to 8.21 μg/day (a decrease of 32%) for TEQ (Table 3). In the case of large WTPs, a 10% decrease of total PCDD loads was observed (from 335.45 to 370.27 μg/day), whereas significantly greater reductions were observed for PCDF and TEQ loads, reaching 67 (a decrease from 973.56 to 320.23 μg/day) and 70% (a decrease from 180.06 to 53.82 μg/day), respectively, between high and stable flow (Table 3).

The obtained results show that generally, during stable wastewater flow, WTPs released smaller PCDD, PCDF, and TEQ loads into the recipients. This is related to both the lower volume of treated wastewater (see Table 1) and the lower concentrations of the analyzed compounds in the effluent collected during periods of stable hydrology (see Table 2).

## Conclusions

The WTPs represent an obligatory and final step prior to the release of wastewater into the environment. Hence, an emerging task for WTPs would be to act as a barrier for micropollutants, preventing the emission of potentially harmful substances into the aqueous environment. Nevertheless, WTPs are widely recognized as an important source of toxic contaminants such as PCDDs/Fs. An example is given in the present paper, where the concentrations of 17 2,3,7,8-substituted PCDD/F congeners were investigated in effluents from 14 WTPs of different sizes and under conditions of high and stable wastewater flow.

The results reveal that the studied WTPs did not purify the wastewater sufficiently. In all the samples, toxic congeners of PCDD/F were detected, with PCDFs prevailing over PCDDs and the highest concentrations being noted for the smallest WTPs. As their wastewater outflow rates were tens of times higher than smaller WTPs, the largest WTPs discharged much higher loads of the analyzed compounds, despite having the lowest total and TEQ concentrations. Hence, they may deteriorate the quality of the receiving river water to a much greater extent.

Moreover, the study shows the impact of hydrological conditions with regard to high and stable wastewater flow on PCDD/F concentrations. Elevated TEQ values, and hence a lower quality of treated wastewater, were observed at high flow due to reduced treatment efficiency.

The results obtained herein, together with previous literature data, indicate the need to better understand the fate of PCDDs/Fs during the wastewater treatment process and to more precisely quantify the efficiency of the purification methods. This better understanding will allow more effective methods for their removal to be further developed and applied.

## Electronic supplementary material

Tables 1S (DOCX 27 kb)
