# Supplementary material for: The variability of PCDD/F concentrations in the effluent of wastewater treatment plants with regard to their hydrological environment
Source: Environ Monit Assess. 2017 Jan 31;189(2):90. doi: 10.1007/s10661-017-5794-9 (PMC5285434; doi:10.1007/s10661-017-5794-9)
Supplement: Supplementary file 1 — (DOCX 27 kb) [file 10661_2017_5794_MOESM1_ESM.docx]

**Table 1S.** Percentage compositions of PCDD/PCDF congeners in wastewater effluent at high and stable wastewater flow.

| **WTP size** | **Small WTPs** | | | | | | | **Medium WTPs** | | | **Large WTPs** | | | |
| --- | --- | --- | --- | --- | --- | --- | --- | --- | --- | --- | --- | --- | --- | --- |
| **Congener** | **Koniecpol** | **Rozprza** | **Spała** | **Wielgomłyny** | **Gorzkowice** | **Wolbórz** | **Ujazd** | **Tuszyn** | **Sulejów** | **Nowe Miasto** | **Opoczno** | **Warka** | **Tomaszów Mazowiecki** | **Piotrków Trybunalski** |
| **High wastewater flow** | | | | | | | | | | | | | | |
| **2378-TCDD** | 2.42 | 2.67 | 2.35 | 2.07 | 2.64 | 2.54 | 2.53 | 2.43 | 2.21 | 2.50 | 2.20 | 2.56 | 2.54 | 3.57 |
| **12378-PeCDD** | 3.32 | 3.41 | 3.04 | 2.62 | 3.40 | 3.18 | 3.37 | 3.68 | 3.12 | 3.18 | 2.98 | 3.10 | 3.90 | 3.29 |
| **123478-HxCDD** | 5.24 | 4.85 | 5.51 | 4.02 | 4.88 | 5.20 | 5.02 | 6.43 | 4.93 | 4.92 | 5.21 | 4.71 | 5.13 | 4.86 |
| **123678-HxCDD** | 2.50 | 2.60 | 2.41 | 2.02 | 2.60 | 2.17 | 2.42 | 4.27 | 2.69 | 2.61 | 2.16 | 2.62 | 2.23 | 1.95 |
| **123789-HxCDD** | 4.05 | 4.05 | 3.87 | 3.22 | 4.04 | 3.55 | 3.76 | 4.83 | 3.97 | 4.05 | 3.54 | 3.97 | 3.77 | 3.24 |
| **1234678-HpCDD** | 3.93 | 3.50 | 3.60 | 3.07 | 3.68 | 3.58 | 3.94 | 5.31 | 7.65 | 3.51 | 3.69 | 3.43 | 3.54 | 3.05 |
| **OCDD** | 4.42 | 2.89 | 2.85 | 2.25 | 2.52 | 3.44 | 3.06 | 4.51 | 2.67 | 2.50 | 4.54 | 2.42 | 2.87 | 8.05 |
| **2378-TCDF** | 3.93 | 4.76 | 4.32 | 3.47 | 4.08 | 3.98 | 3.55 | 2.99 | 3.41 | 4.09 | 3.76 | 4.31 | 4.23 | 9.43 |
| **12378-PeCDF** | 4.50 | 5.08 | 4.62 | 3.87 | 5.08 | 4.71 | 4.50 | 3.79 | 4.27 | 5.21 | 4.25 | 5.18 | 4.79 | 4.95 |
| **23478-PeCDF** | 7.08 | 7.78 | 8.41 | 6.04 | 7.79 | 8.92 | 7.87 | 6.64 | 6.40 | 8.07 | 8.19 | 8.41 | 8.77 | 7.76 |
| **123478-HxCDF** | 8.15 | 8.48 | 9.24 | 7.07 | 8.51 | 8.80 | 7.94 | 6.70 | 7.04 | 9.08 | 8.50 | 9.49 | 8.97 | 6.76 |
| **123678-HxCDF** | 7.20 | 7.52 | 6.36 | 5.79 | 7.63 | 6.64 | 6.96 | 5.87 | 6.64 | 7.46 | 6.13 | 7.60 | 6.64 | 6.05 |
| **234678-HxCDF** | 9.01 | 8.68 | 8.69 | 7.29 | 8.95 | 8.20 | 8.29 | 6.99 | 9.84 | 8.51 | 7.87 | 8.48 | 7.84 | 7.00 |
| **123789-HxCDF** | 7.37 | 8.10 | 7.58 | 6.32 | 7.83 | 8.29 | 8.12 | 6.85 | 6.42 | 7.89 | 8.33 | 7.81 | 7.41 | 6.76 |
| **1234678-HpCDF** | 9.13 | 9.03 | 8.30 | 7.29 | 8.95 | 8.31 | 9.38 | 7.91 | 8.53 | 9.63 | 8.61 | 9.69 | 9.25 | 7.29 |
| **1234789-HpCDF** | 9.50 | 9.16 | 10.21 | 8.64 | 9.75 | 9.87 | 10.01 | 8.45 | 12.10 | 9.19 | 10.88 | 8.82 | 9.41 | 8.29 |
| **OCDF** | 8.27 | 7.46 | 8.63 | 24.94 | 7.67 | 8.63 | 9.28 | 12.36 | 8.10 | 7.60 | 9.18 | 7.40 | 8.72 | 7.71 |
| **Stable wastewater flow** | | | | | | | | | | | | | | |
| **2378-TCDD** | 3.44 | 1.69 | 2.00 | 2.33 | 0.84 | 0.13 | 2.27 | 2.14 | 2.71 | 3.45 | 2.22 | 2.13 | 2.87 | 0.81 |
| **12378-PeCDD** | 3.91 | 1.95 | 2.40 | 2.84 | 1.08 | 0.31 | 2.57 | 2.92 | 3.07 | 3.94 | 2.22 | 2.66 | 3.45 | 0.90 |
| **123478-HxCDD** | 5.00 | 3.23 | 4.40 | 4.91 | 3.36 | 0.27 | 4.94 | 4.35 | 5.10 | 5.42 | 3.48 | 4.79 | 5.17 | 1.42 |
| **123678-HxCDD** | 2.44 | 1.74 | 2.00 | 2.58 | 1.20 | 0.15 | 2.27 | 2.21 | 2.51 | 2.46 | 9.49 | 2.13 | 2.87 | 1.57 |
| **123789-HxCDD** | 7.45 | 2.36 | 3.20 | 3.62 | 2.04 | 0.20 | 3.30 | 3.11 | 3.98 | 4.43 | 2.85 | 3.19 | 4.02 | 1.04 |
| **1234678-HpCDD** | 2.83 | 2.46 | 3.60 | 4.13 | 6.24 | 13.27 | 3.09 | 2.53 | 3.11 | 3.94 | 2.85 | 3.72 | 3.45 | 6.17 |
| **OCDD** | 4.33 | 31.35 | 12.40 | 3.36 | 45.62 | 62.81 | 10.30 | 2.14 | 5.97 | 3.45 | 11.39 | 9.04 | 2.87 | 59.35 |
| **2378-TCDF** | 5.12 | 2.36 | 4.40 | 3.62 | 1.20 | 0.98 | 5.46 | 3.70 | 3.98 | 3.45 | 12.66 | 3.72 | 3.45 | 3.47 |
| **12378-PeCDF** | 4.89 | 3.44 | 4.00 | 4.39 | 1.56 | 0.58 | 3.81 | 4.09 | 4.78 | 4.43 | 3.16 | 4.26 | 4.60 | 1.38 |
| **23478-PeCDF** | 7.77 | 5.23 | 6.40 | 7.49 | 2.52 | 2.63 | 6.59 | 11.36 | 7.56 | 6.40 | 5.06 | 6.91 | 8.05 | 2.85 |
| **123478-HxCDF** | 8.45 | 5.70 | 7.60 | 8.27 | 3.72 | 1.56 | 8.34 | 6.75 | 8.44 | 7.39 | 6.65 | 7.45 | 9.20 | 2.71 |
| **123678-HxCDF** | 6.24 | 4.46 | 5.60 | 6.46 | 3.60 | 1.60 | 5.87 | 5.52 | 7.17 | 5.91 | 4.43 | 5.85 | 6.90 | 1.90 |
| **234678-HxCDF** | 7.06 | 5.28 | 7.20 | 8.27 | 4.80 | 0.67 | 8.24 | 7.59 | 7.72 | 9.36 | 5.70 | 7.98 | 8.05 | 2.61 |
| **123789-HxCDF** | 6.74 | 4.93 | 6.40 | 7.24 | 2.76 | 1.60 | 7.11 | 5.84 | 7.32 | 7.39 | 5.38 | 7.45 | 7.47 | 2.18 |
| **1234678-HpCDF** | 8.21 | 6.31 | 8.80 | 9.82 | 7.44 | 5.35 | 8.24 | 22.45 | 9.28 | 9.85 | 6.65 | 9.57 | 9.20 | 3.32 |
| **1234789-HpCDF** | 7.62 | 11.13 | 9.60 | 10.34 | 3.48 | 0.62 | 8.34 | 6.75 | 8.64 | 10.34 | 8.23 | 10.11 | 9.20 | 2.71 |
| **OCDF** | 8.51 | 6.36 | 10.00 | 10.34 | 8.52 | 7.26 | 9.27 | 6.55 | 8.68 | 8.37 | 7.59 | 9.04 | 9.20 | 5.60 |
